# Childhood Hemolytic Uremic Syndrome, United Kingdom and Ireland

**DOI:** 10.3201/eid1104.040833

**Published:** 2005-04

**Authors:** Richard M. Lynn, Sarah J. O'Brien, C. Mark Taylor, Goutam K. Adak, Henrik Chart, Tom Cheasty, John E. Coia, Iain A. Gillespie, Mary E. Locking, William J. Reilly, Henry R. Smith, Aoife Waters, Geraldine A. Willshaw

**Affiliations:** * Royal College of Paediatrics and Child Health, London, United Kingdom;; † Communicable Disease Surveillance Centre, London, United Kingdom;; ‡ Birmingham Children's Hospital, Birmingham, United Kingdom;; § Health Protection Agency Centre for Infections, London, United Kingdom;; ¶ Western General Hospital, Edinburgh, United Kingdom;; # Scottish Centre for Infection and Environmental Health, Glasgow, United Kingdom;; ** The Children's Hospital, Dublin, Ireland

**Keywords:** Morbidity, mortality, zoonoses, pediatrics, population surveillance, hemolytic uremic syndrome, diarrhea, Escherichia coli O157, United Kingdom, Ireland, gastroenteritis, research

## Abstract

We conducted prospective surveillance of childhood hemolytic uremic syndrome (HUS) from 1997 to 2001 to describe disease incidence and clinical, epidemiologic and microbiologic characteristics. We compared our findings, where possible, with those of a previous study conducted from 1985 to 1988. The average annual incidence of HUS for the United Kingdom and Ireland (0.71/100,000) was unchanged from 1985 to 1988. The overall early mortality had halved, but the reduction in mortality was almost entirely accounted for by improved outcome in patients with diarrhea-associated HUS. The principal infective cause of diarrhea-associated HUS was Shiga toxin–producing *Escherichia coli* O157 (STEC O157), although in the 1997–2001 survey STEC O157 phage type (PT) 21/28 had replaced STEC O157 PT2 as the predominant PT. The risk of developing diarrhea-associated HUS was significantly higher in children infected with STEC O157 PT 2 and PT 21/28 compared with other PTs. Hypertension as a complication of HUS was greatly reduced in patients with diarrhea-associated HUS.

## Abstract 2

The risk for diarrhea-associated HUS was higher for children infected with *Escherichia coli* O157 phage type (PT) 2 and PT21/28 than for those infected with other PTs.

The most serious manifestation of infection with Shiga toxin–producing *Escherichia coli* (STEC) in humans is hemolytic uremic syndrome (HUS). This syndrome comprises a triad of microangiopathic hemolytic anemia, thrombocytopenia, and acute renal failure, usually after a prodromal illness of acute gastroenteritis (1). At least 80% of childhood HUS is attributable to infection with STEC (2), mainly serogroup O157, although other serogroups are implicated (2–10). The peak incidence of HUS is in children <5 years of age (1). Surveillance of pediatric HUS provides valuable information on human infection with STEC.

In a prospective survey of pediatric HUS from 1985 to 1988 in the British Isles, the average annual incidence was ≈0.79 per 100,000 children <16 years of age (3). In the intervening years, the number of laboratory-confirmed cases of STEC O157 in England and Wales increased from 50 in 1985 (11) to 1,087 in 1997 (12). Similar increases were seen in Scotland and Ireland (Figure). Some of the increase in STEC O157 might reflect improved laboratory techniques, improved detection or reporting of milder cases, and a greater awareness of the need to investigate diarrheal disease for STEC. If the increase in STEC O157 was real, however, then the incidence of childhood HUS should have also increased. Therefore, we conducted prospective surveillance of childhood HUS in the United Kingdom and Ireland from 1997 to 2001 to describe the impact of disease and clinical, epidemiologic, and microbiologic characteristics of the patients. We also compared our findings, where possible, with those of a previous study (3,4).

**Figure:**
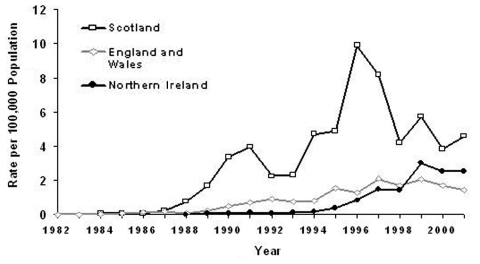
Laboratory-confirmed infection with Shiga toxin–producing *Escherichia coli* O157 in the United Kingdom, 1982–2001. Data sources: Public Health Laboratory Service and Scottish Center for Infection and Environmental Health.

## Patients and Methods

### Definition of HUS

HUS was defined as 1) acute renal failure, including oligoanuria and elevated creatinine level for age, 2) microangiopathic hemolytic anemia (hemoglobin level <10 g/L with fragmented erythrocytes), and 3) thrombocytopenia (platelet count <130,000 × 10^9^/L) in the absence of septicemia, malignant hypertension, chronic uremia, or primary vascular disease. Diarrhea-associated HUS was defined as disease that immediately followed diarrhea or bloody diarrhea. Nondiarrhea-associated HUS was defined as disease in which ≥1 episodes did not follow an episode of diarrhea.

### Case Ascertainment

Prospective, active surveillance of children <16 years of age was undertaken from February 1, 1997, to January 31, 2001, in the United Kingdom and Ireland. Pediatricians actively reported new cases of HUS in children to the British Paediatric Surveillance Unit or the Scottish Centre for Infection and Environmental Health, by using a card mailed to them each month (13,14). This card allowed them to indicate how many new cases of HUS they had diagnosed. Physicians were also encouraged to telephone the Public Health Laboratory Service Communicable Disease Surveillance Centre or Scottish Centre for Infection and Environmental Health to expedite the identification and investigation of localized outbreaks.

### Clinical and Epidemiologic Data Collection

After a report was submitted to the British Paediatric Surveillance Unit, a short, standard, structured questionnaire was mailed to the reporting clinician to collect basic epidemiologic data that included family history, clinical features, laboratory investigations, and initial outcome. Outcome information was requested both on the initial questionnaire, and if unclear, on a follow-up questionnaire ≈1 year from onset. Full renal recovery was defined as normal blood pressure and creatinine level for age and the absence of proteinuria on strip reagent urinalysis. Reminders were sent at the end of the study period to those clinicians who did not return the questionnaire within a month and to any nonresponders.

### Microbiologic Investigations

Clinicians were asked to obtain fecal and serum samples from all patients with HUS (both with and without diarrhea) according to a standard clinical protocol. Fecal samples were examined for *E. coli* O157 at the local microbiology laboratory. Presumptive *E. coli* O157 isolates were referred to the Laboratory of Enteric Pathogens at Colindale or the Scottish *E. coli* O157 Reference Laboratory in Edinburgh (in Aberdeen until April 1999). Fecal specimens were obtained from some patients from whom *E. coli* O157 had not been isolated. Serum samples were also referred to the reference laboratories.

Presumptive *E. coli* O157 isolates were confirmed biochemically as *E. coli* and as O157 by serotyping. Confirmed *E. coli* O157 isolates were phage typed and tested for *stx* genes by DNA hybridization or polymerase chain reaction (PCR). Fecal specimens referred to the reference laboratories were examined for *E. coli* O157 by using immunomagnetic separation and also for all STEC by PCR and DNA hybridization (12). Serum samples were tested for antibodies to *E. coli* O157 lipopolysaccharide (15,16). Methods for examination of isolates and fecal samples sent to the reference laboratories from other cases of STEC infection during the study period have been described previously (12).

The distribution of phage types (PTs) of STEC O157 in patients with HUS was compared with that of all cases of infection with STEC O157 in children <16 years of age in England, Wales, and Scotland (comparable data for Northern Ireland and Ireland were not available).

### Data Validation and Removal of Duplicate Data

Duplicate reports for the same patient were possible since cases might be reported both from district general hospitals and from specialist pediatric nephrology units. Duplicates were removed by using full name and date of birth. Where duplicates were identified, reports from a specialist in pediatric nephrology were used in preference to those from district general hospitals. The microbiologic and questionnaire data were entered into a Microsoft (Redmond, WA, USA) Access database and linked by using full name and date of birth.

### Ethical Approval

This study was reviewed and approved by the Ethics Committee of the Public Health Laboratory Service and the Ethics Committee of the South Birmingham Health Authority.

### Data Analysis

Incidence rates and 95% confidence intervals (CIs) were calculated by using population denominators obtained from the National Statistics Office (for the United Kingdom) and the Central Statistics Office (for Ireland). Descriptive and statistical analyses were performed in Microsoft Access and Excel and in STATA version 7 (Stata Corp., College Station, TX, USA). Differences in proportions were assessed with the chi-square test, and means were compared with the Z test.

## Results

### Response Rate

During the 4-year period of surveillance, 92% of pediatricians responded to the active reporting system. Four hundred thirteen cases of HUS were confirmed, of which 218 occurred in girls. Three hundred ninety-five patients had diarrhea-associated HUS, and 18 had nondiarrhea-associated HUS.

### Incidence

Although most of the HUS cases were reported from England, the highest incidence rates were in Scotland (Table 1). The average annual incidence for those <5 years of age in Scotland was significantly higher than that in England (risk ratio 2.28, 95% CI 1.63–2.30, p < 0.001) (Table 1). The age and sex distributions are also shown in Table 1.

**Table 1:** Number, sex distribution, and incidence of childhood cases of hemolytic uremic syndrome, United Kingdom and Ireland, 1997–2001

	Year											
	1	2	3	4	Males	Females	Total	Incidence per 10^5^/y (all cases)	95% CI*	Patient, <5 y of age	Incidence per 10^5^/y (patients <5 y)	95% CI*
England	83	66	65	73	139	148	287	0.71	0.56–0.89	185	1.54	1.12–2.0
Scotland	12	19	20	12	28	35	63	1.56	0.9–2.43	41	3.4	1.84–6.17
Wales	3	5	4	5	5	12	17	0.71	0.27–1.70	10	1.49	0.36–4.18
Northern Ireland	2	6	4	4	9	7	16	0.97	0.27–1.70	7	1.45	0.2–4.6
Ireland	3	7	10	10	14	16	30	0.83	0.38–1.6	24	2.33	0.85–5.1
Total	103	103	103	104	195	218	413	0.71	0.56–0.89	267	1.54	1.12–2.0

*CI, confidence interval.

### Clinical Features and Complications

The clinical features and complications for children from the 1997–2001 survey are summarized in Table 2 and compared with those of the HUS cases from the 1985–1988 survey (3,4). The proportions of HUS patients with and without a diarrheal prodome were very similar in the 2 surveys. The mean duration of illness from symptom onset to diagnosis of HUS decreased, and smaller proportions of children with diarrhea-associated HUS in the 1997–2001 survey showed severe acute abdominal symptoms or hypertension. HUS was reported in 20 pairs of siblings and, in 4 instances, between cousins, with the index patient in each incident having had a diarrheal prodrome.

**Table 2:** Clinical features and acute complications of diarrhea-associated hemolytic uremic syndrome (HUS) in children from the 1997–2001 British Paediatric Surveillance Unit survey compared with children from the 1985–1988 survey

	1985–1988 survey	1997–2001 survey	p value
No. of cases	288	413	0.6
Cases with a diarrheal prodrome	273 (95%)	395 (96%)	
Mean (range) time from onset of diarrhea to diagnosis of HUS	8 days (1–34)	6 days (range 1–35)	<0.001
Severe acute abdominal symptoms	40 (15%)	36 (9%)	0.03
Seizures or other neurologic complications	51(19%)	52 (13%)	0.06
Hypertension	86 (32%)	92 (23%)	0.02
Cardiomyopathy	4 (1%)	7 (2%)	1.0
Diabetes mellitus	4 (1%)	8 (2%)	0.77

Of the 18 children with nondiarrhea-associated HUS, 1 developed HUS during treatment for acute lymphoblastic leukemia. The clinical course was complicated by pancreatitis, diabetes mellitus, and seizures. Six children had recurrent HUS, although only 3 had recurrences during the surveillance period. Five of these children had 1 episode of diarrhea-associated HUS (bloody diarrhea in 3 cases). However, all 6 children had relapses without diarrhea and were considered cases of nondiarrhea-associated HUS. All 6 children were negative for STEC.

### Treatment

Sixty-three patients with diarrhea-associated HUS received antimicrobial agents (penicillin, metronidazole, or a second- or third-generation cephalosporin) before admission. Eight patients received ciprofloxacin. No statistically significant difference in outcome was seen for those receiving antimicrobials agents (data not shown).

### Clinical Outcome

Eighty-two percent of the patients were treated in specialist pediatric nephrology centers. Outcome data were available for 389 (98%) with diarrhea-associated HUS and 18 (100%) with nondiarrhea-associated HUS. Seven (1.8%) children with diarrhea-associated HUS died during acute illness compared with 14 (5.6%) of 252 (χ^2^ = 6.8, p = 0.009) in the earlier survey. The early death rate for diarrhea-associated HUS was 22% (4/18) from 1997 to 2001 compared with 21% (4/14) from 1985 to 1988 (p = 0.7, Fisher exact test). The overall early death rate for the combined group was 2.5% compared with 5% in the 1985–1988 survey (χ^2^ = 6.8, p = 0.01). Eight of the 11 deaths in the 1997–2001 survey were in children <5 years of age. In the group treated with antimicrobial agents, 3 deaths (5%) occurred compared with 4 deaths (1.2%) in the group that was not treated. This difference was not significant, and no significant relationship was seen when the outcomes of death and renal impairment were analyzed together (data not shown).

Renal recovery was reported in 342 (88%) of those with diarrhea-associated HUS compared with 10 (56%) of those with nondiarrhea-associated HUS (p = 0.001, Fisher exact test). Hypertension was more likely to develop in patients with diarrhea-associated HUS than those with nondiarrhea-associated HUS (p = 0.05). Seizures or other neurologic complications (p = 0.008), and death (p = 0.0007) were also more likely to occur in those with diarrhea-associated HUS.

### Microbiologic Findings for Fecal Samples and Sera

Stools and or sera were available for testing from 393 of 395 patients with diarrhea-associated HUS. Only 25 fecal samples from which presumptive *E. coli* O157 was not isolated locally were examined for the presence of STEC and non-O157 STEC by the reference laboratories. Shiga toxin–producing *E. coli* O157 was confirmed in 10, and 1 was positive for STEC O26.

The proportion of cases confirmed through fecal sampling or serology was much greater (84%) than in 1985–1988, when only 31% of cases were laboratory confirmed (p < 0.001). In the 1997–2001 survey, 161 (60%) of 270 serum samples from patients with diarrhea-associated HUS were positive for antibodies to *E. coli* O157 lipopolysaccharide. Combining results from fecal sampling and serology showed that 329 (83%) of 395 patients with diarrhea-associated HUS were infected with STEC O157; 1 was infected with STEC O26. A total of 330 (84%) patients with diarrhea-associated HUS were infected with STEC.

In the remaining 65 patients with diarrhea-associated HUS, no infective agents were found in 59, *Campylobacter* was found in 2, *Shigella sonnei* PT12 in 1, *Streptococcus pneumoniae* in 1, group C *Streptococcus* in 1, and *Staphylococcus aureus* in 1. In the STEC-positive patients, coinfections with *Campylobacter* (3 patients), group B *Streptococcus* (1 patient), *Cryptosporidium* (3 patients), *Salmonella* (3 patients), and rotavirus (1 patient) were identified.

Eight patients had evidence of infection with *S. pneumoniae*, without other infections including STEC; 7 did not have diarrhea. Two patients were girls and 6 were boys. Three patients had hypertension, 2 showed signs of hemorrhage, and 4 had seizures. Five of the 8 patients made a full renal recovery, but 1 has permanently impaired renal function, and 2 died.

### Properties of STEC O157 Strains

The properties of STEC strains belonging to serogroup O157 are summarized in Table 3. Phage typing results were available for 220 (84%) of 261 STEC O157 isolates from patients with diarrhea-associated HUS. The risk of developing this type of HUS was significantly higher for children infected with STEC O157 PT21/28 and STEC O157 PT2.

**Table 3:** Properties of STEC strains of *Escherichia coli* serogroup O157 from patients <16 years of age with HUS with and without diarrhea compared with STEC O157 strains from all infected children <16 years of age, 1997–2001*

Phage type	STEC-infected cases with HUS with diarrhea	STEC-infected cases with HUS without diarrhea	All STEC-infected cases	%	Relative risk versus all other phage types (95% CI)
England, Wales, and Scotland					
2	86	537	623	14	1.59 (1.23–2.05)
21/28	101	592	693	15	1.80 (1.41–2.31)
8	1	236	237	0.4	0.04 (0.005–0.26)
32	4	159	163	2	0.22 (0.09–0.6)
4	9	126	135	7	0.64 (0.34–1.22)
Others	19	296	315	6	–
Total	220	1,946	2,166	10	–
England and Wales					
2	77	475	552	14	1.72 (1.3–2.28)
21/28	67	357	424	16	1.96 (1.47–2.6)
8	1	209	210	0.5	0.04 (0.006–0.3)
32	3	145	148	2	0.19 (0.06–0.58)
4	8	118	126	6	0.62 (0.31–1.23)
Others	16	248	264	6	–
Total	172	1,552	1,724	10	–
Scotland					
2	9	62	71	13	1.21 (0.61–2.38)
21/28	34	235	269	13	1.56 (0.86–2.82)
8	0	27	27	0	–
32	1	14	15	7	0.61 (0.09–4.1)
4	1	8	9	11	1.02 (0.16-6.62)
Others	3	48	51	6	–
Total	48	394	442	11	–

*STEC, Shiga toxin–producing *Escherichia coli*; HUS, hemolytic uremic syndrome; CI, confidence interval.

Overall the STEC O157 PTs that predominated were PT21/28 and PT2. However, geographic differences were striking: PT2 was dominant in England and Wales, PT21/28 in Scotland, and PT32 (6/10) in Ireland. Patients in Scotland with diarrhea-associated HUS were less likely to be infected with STEC O157 PT2 than those in England and Wales (χ^2^ = 10.62, p = 0.001) but were more likely to be infected with PT21/28 (χ^2^ = 14.72, p = 0.0001). In the 1985–1988 survey, PT2 was dominant (25/38, 66%) and PT21/28 was not found.

Data on Shiga toxin typing were available for 220 strains. Most (213/220, 97%) had only *stx* 2 genes and 7 (3%) had *stx* 1 + 2 genes.

## Discussion

In this survey, the average annual incidence of HUS for the United Kingdom and Ireland from 1997 to 2001 was unchanged from the incidence from 1985 to 1988, despite large increases in laboratory-confirmed cases of STEC O157 infection. This finding probably reflects underdiagnosis of STEC infection. Similar proportions of cases with diarrhea-associated and nondiarrhea-associated HUS were found, but the proportion with a confirmed diagnosis had increased. As in the 1985–1988 survey, the principal cause of HUS was STEC O157, but in the 1997–2001 study STEC O157 PT21/28 had replaced STEC O157 PT2 as the predominant PT. This finding reflected the emergence of PT21/28 first in Scotland and subsequently in England and Wales (17,18). However, despite the overall dominance of PT21/28, geographic differences in PT distribution occurred across the United Kingdom and Ireland.

The illness and death rate of patients with diarrhea-associated HUS remained high. These children were younger than those with nondiarrhea-associated HUS caused by STEC and had significantly poorer outcomes. Hypertension as a complication of HUS was greatly reduced among patients with diarrhea-associated HUS. The overall death rate had halved, but the reduction in deaths was almost entirely accounted for by improved outcomes in these cases.

The 1985–1988 and 1997–2001 surveys were similar in most regards. Acquisition of data was identical, and the same diagnostic criteria for HUS were used. However, a difference in microbiologic investigation of stool samples was seen in the 2 surveys. In the 1985–1988 survey, fresh fecal samples were sent directly to the Public Health Laboratory Service Division of Enteric Pathogens for culturing and complete strain identification directly from clinical samples. In the 1997–2001 survey, the initial diagnostic work was undertaken in local microbiology departments, and presumptive isolates were forwarded to the appropriate reference laboratories for confirmation and complete identification. The only clinical samples forwarded to the reference laboratories were some of those from HUS patients who were negative on examination at a local laboratory. Although all facilities in the Public Health Laboratory Service had been using a standard protocol since 1995, following a recommendation of the Advisory Committee on the Microbiological Safety of Food that all diarrheal samples be tested for STEC O157 (11,19), this testing was not done at all National Health Service laboratories. A potential criticism of this study is that non-O157 STEC strains might have been missed. This omission is unlikely because 83% of cases had evidence of *E. coli* O157 infection. Little testing for non-O157 STEC occurs in primary diagnostic laboratories in the United Kingdom. However, this situation needs to be balanced against the fact that involvement of local laboratories in assisting clinical investigations expedited fecal sample testing, as shown by a marked increase in sampling, coupled with an improved diagnostic yield. The proportion of culture-positive specimens, which was more than twice as high as in the previous survey, was also higher than proportions reported by other investigators (2,6,9,10). In the 1997–2001 survey, the additional benefit of serologic testing for antibodies to *E. coli* O157 as a diagnostic tool was evident.

A combination of increasing diagnostic yield and shortening time between onset of diarrhea and diagnosis of infection with STEC O157 should allow clinicians to monitor patients and intervene earlier should signs of renal involvement occur (20,21). Presumably, the significant reduction in time to diagnosis is a function of increasing awareness of STEC O157 infection and its complications among clinicians and microbiologists. This finding might explain the reduction in hypertension and deaths in patients with diarrhea-associated HUS through earlier intervention, including management of dehydration in children before onset of HUS, which has been shown to improve outcome (20). Although the reduction in neurologic complications of diarrhea-associated HUS was not significant, it might be clinically important.

STEC O157 remains the dominant cause of HUS in the United Kingdom and Ireland, with other serotypes contributing little to the overall impact of disease. This scenario is similar to the situation in the United States (2), but contrasts sharply with that in Australia, where infection with STEC O157 is rare (7). In mainland Europe, STEC O157 is the most common cause of HUS, but the contribution of other serotypes is also important (5,6,8–10). The number of cases of HUS not caused by STEC O157 in the 1997–2001 survey was small and much lower than in the previous survey. Eighty-five percent of all O157 strains isolated from children with HUS had either of 2 PTs: PT2 and PT21/28. STEC O157 PT 21/28 emerged during this study and was first seen in Scotland, appearing in England and Wales 2 years later (17,18). Compared with all the STEC O157 strains in children <16 years of age typed by the reference laboratories, PT2 and PT21/28 strains were overrepresented in the patients with HUS, suggesting that these strains might have specific virulence in the children. Most of the PT2 and PT21/28 strains produced *stx*2 either alone or in combination with *stx*1. Shiga toxin type 2 is generally considered an important virulence factor in the pathogenesis of HUS (22,23).

HUS is the most important clinical effect of STEC infection in humans, and young children are more vulnerable than any other age group. It follows that surveillance of childhood HUS is a valuable tool for monitoring the effect of STEC in a population and provides early warning of change. The diagnosis of HUS is obvious and unambiguous, and changes in the incidence of the condition are readily detected and meaningful. Moreover, by focusing on a small indicator population, we observed that this method of surveillance is relatively inexpensive and efficient.

The incidence of HUS in the 1997–2001 survey was similar to that found in 1985–1988 (3). This finding suggests that the incidence of clinically relevant STEC infection has remained constant, at least in children. Therefore, the increase in laboratory reporting over the same time implies increased awareness and readiness to investigate diarrhea or illness by using appropriate microbiologic techniques. More laboratory testing and improved reporting might indicate that milder cases of disease are recognized, so that the proportion of cases of infection with STEC leading to HUS has decreased. Alternatively, improving the management of diarrhea-associated HUS through an earlier diagnosis might allow clinicians to intervene earlier in the disease process, as demonstrated by reductions in hypertension and deaths. This means that the course of the disease might have been altered, explaining, at least in part, the unchanged overall incidence.

## References

[1] Fitzpatrick M. Haemolytic uraemic syndrome and *E. coli* O157. BMJ. 1999;318:684–5. 10.1136/bmj.318.7185.684 10073994 PMC1115131

[2] Banatvala N, Griffin PM, Greene KD, Barrett TJ, Bibb WF, Green JH, The United States National Prospective Hemolytic Uremic Syndrome Study: microbiologic, serologic, clinical, and epidemiologic findings. J Infect Dis. 2001;183:1063–70. 10.1086/319269 11237831

[3] Milford DV, Taylor CM, Guttridge B, Hall SM, Rowe B, Kleanthous H. Haemolytic uraemic syndromes in the British Isles 1985–8: association with verocytotoxin producing *Escherichia coli*. Part 1: clinical and epidemiological aspects. Arch Dis Child. 1990;65:716–21. 10.1136/adc.65.7.716 2201261 PMC1792437

[4] Kleanthous H, Smith HR, Scotland SM, Gross RJ, Rowe B, Taylor CM, Haemolytic uraemic syndromes in the British Isles, 1985–8: association with verocytotoxin producing *Escherichia coli*. Part 2: microbiological aspects. Arch Dis Child. 1990;65:722–7. 10.1136/adc.65.7.722 2201262 PMC1792459

[5] Gerber A, Karch H, Allerberger F, Verweyen HM, Zimmerhackl LB. Clinical course and the role of shiga toxin–producing *Escherichia coli* infection in the hemolytic-uremic syndrome in pediatric patients, 1997–2000, in Germany and Austria: a prospective study. J Infect Dis. 2002;186:493–500. 10.1086/341940 12195376

[6] Tozzi AE, Caprioli A, Minelli F, Gianviti A, De Petris L, Edefonti A, Shiga toxin–producing *Escherichia coli* infections associated with hemolytic uremic syndrome, Italy, 1988–2000. Emerg Infect Dis. 2003;9:106–8. 12533290 10.3201/eid0901.020266 PMC2873761

[7] Elliott EJ, Robins-Browne RM, O'Loughlin EV, Bennett-Wood V, Bourke J, Henning P, Nationwide study of haemolytic uraemic syndrome: clinical, microbiological, and epidemiological features. Arch Dis Child. 2001;85:125–31. 10.1136/adc.85.2.125 11466187 PMC1718875

[8] Scheutz F. The significance of non-O157 VTEC infections. [monograph on the Internet]. [cited 23 Dec 2003]. Available from http://www.vtec-edinburgh.com/docs/Vtec-Final-text.pdf

[9] Cornu G, Proesmans W, Dediste A, Jacobs F, Van De Walle J, Mertens A, Hemolytic uremic syndrome in Belgium: incidence and association with verocytotoxin-producing *Escherichia coli* infection. Clin Microbiol Infect. 1999;5:16–22. 10.1111/j.1469-0691.1999.tb00093.x 11856208

[10] Haeghebaert S, Vaillant V, Decludt B, Bouvet P, Grimont PA. Surveillance of haemolytic uraemic syndrome in children under 15 years of age in France in 1998. Eurosurveillance. 2000;5:68–73. 12631856 10.2807/esm.05.06.00032-en

[11] Advisory Committee on the Microbiological Safety of Food. Report on verocytotoxin-producing *Escherichia coli*. London: Her Majesty's Stationery Office; 1995.

[12] Willshaw GA, Cheasty T, Smith HR, O'Brien SJ, Adak GK. Verocytotoxin-producing *Escherichia coli* (VTEC) O157 and other VTEC from human infections in England and Wales: 1995–1998. J Med Microbiol. 2001;50:135–42. 11211220 10.1099/0022-1317-50-2-135

[13] Verity C, Preece M. Surveillance for rare disorders by the BPSU. Why is it worthwhile? Arch Dis Child. 2002;87:269–71. 10.1136/adc.87.4.269 12243988 PMC1763054

[14] Adak GK, Lynn R, O'Brien SJ. HUS surveillance: What does it tell us about VTEC? SCIEH Wkly Rep. 2000;34(Suppl):14.

[15] Chart H, Smith HR, Scotland SM, Rowe B, Milford DV, Taylor CM. Serological identification of *Escherichia coli* O157:H7 infection in haemolytic uraemic syndrome. Lancet. 1991;337:138–40. 10.1016/0140-6736(91)90801-U 1670788

[16] Chart H, Jenkins C. A review: the serodiagnosis of infections caused by verocytotoxin-producing *Escherichia coli.* J Appl Microbiol. 1999;86:731–40. 10.1046/j.1365-2672.1999.00766.x 10347867

[17] Allison L, Taylor P, Hanson M. Genetic subtyping of *Escherichia coli* O157 in Scotland. Fifth International Symposium on Shiga toxin (verocytotoxin)-producing *Escherichia coli* infections. [monograph on the Internet]. [cited 23 Dec 2003]. Available from http://www.vtec-edinburgh.com/docs/Vtec-Final-text.pdf

[18] Smith HR, Cheasty T, Willshaw GA, Caprioli A, Tozzi AE, Coia JE. Changing patterns of VTEC infection in Britain and continental Europe. Ann Ist Super Sanita. 2002;15(Suppl 1):4–6.

[19] Verocytotoxin producing *Escherichia coli*: Which specimens should be tested? CDR Wkly. 1995;5:147. 7670583

[20] Tarr P, Chandler WL, Jelacic S, Wong CS, Ake J, Shaikh N, The pathophysiology of the haemolytic uraemic syndrome: continuing results from a prospective study in the Pacific north west. Fifth International Symposium on Shiga toxin (verocytotoxin)-producing *Escherichia coli* infections. [monograph on the Internet]. [cited 23 Dec 2003]. Available from http://www.vtec-edinburgh.com/docs/Vtec-Final-text.pdf

[21] Dundas S, Todd WT, Stewart AI, Murdoch PS, Chaudhuri AK, Hutchinson SJ. The central Scotland *Escherichia coli* O157:H7 outbreak: risk factors for the hemolytic uremic syndrome and death among hospitalized patients. Clin Infect Dis. 2001;33:923–31. 10.1086/322598 11528561

[22] Ostroff SM, Tarr PI, Neill MA, Lewis JH, Hargrett-Bean N, Kobayashi JM. Toxin genotypes and plasmid profiles as determinants of systemic sequelae in *Escherichia coli* O157:H7 infections. J Infect Dis. 1989;160:994–8. 10.1093/infdis/160.6.994 2685131

[23] Boerlin P, McEwan SA, Boerlin-Petzold F, Wilson JB, Johnson RP, Gyles CL. Association between virulence factors of Shiga toxin-producing *Escherichia coli* and disease in humans. J Clin Microbiol. 1999;37:497–503. 9986802 10.1128/jcm.37.3.497-503.1999 PMC84443

